# Morphological Advantages of Nano-Zinc: Effects on Yield and Quality Improvement in Blue Honeysuckle

**DOI:** 10.3390/plants15101520

**Published:** 2026-05-15

**Authors:** Xuefei Ji, Wei Li, Yuxi Chen, Haihui She, Shan Wang, Chunshuang Li, Hao Sun, Junwei Huo

**Affiliations:** 1 College of Horticulture and Landscape Architecture, Northeast Agricultural University, Harbin 150038, China; jixuefff@163.com (X.J.);; 2 College of Resources and Environment, Northeast Agricultural University, Harbin 150030, China; 3 Harbin Ecology and Agriculture Meteorological Center, Harbin 150028, China; 4 National Key Laboratory of Smart Farm Technologies and Systems, Northeast Agricultural University, Harbin 150030, China

**Keywords:** blue honeysuckle, ZnO NPs, fruit quality, yield

## Abstract

Blue honeysuckle (*Lonicera caerulea* L.) is subject to environmental stressors, leading to variability in both severe fruit drop during development and fruit quality. Zinc, an essential micronutrient, is critical to sustainable fruit tree production by enhancing yield and nutritional quality. Different forms of zinc fertilizers, particularly nano-zinc versus conventional ionic zinc, exhibit marked differences in absorption efficiency and agronomic performance, thereby determining their practical efficacy. In this two-year study, we evaluated the effects of foliar-applied zinc forms, ZnO nanoparticles (30, 50, and 90 nm) and ionic zinc (ZnCl_2_ and ZnSO_4_), applied at the young fruit, veraison, and maturity stages on yield and fruit quality. Results showed that ZnO nanoparticles were more effective than ionic zinc at 80 mg/L. In particular, among the ZnO NP treatments, 90 nm ZnO NPs exhibited the best overall effect, significantly improving fruit quality. The 30 nm ZnO NPs treatment performed best in terms of single fruit weight, yield per plant, and fruit firmness. This study highlights the potential of nano-zinc to enhance productivity and quality in blue honeysuckle, providing a theoretical basis for selecting optimal zinc fertilizer types and particle sizes in specialty berry production, with implications for sustainable, high-quality fruit cultivation.

## 1. Introduction

Blue honeysuckle (*Lonicera caerulea* L.) is a valuable berry crop, mainly distributed in the Greater and Lesser Khingan Mountains and the Changbai Mountain region of northeastern China. As an emerging small berry crop, it has been attracting growing attention [1]. Market demand for blue honeysuckle remains consistently strong; however, its fruit quality is susceptible to climatic and environmental factors, and cultivation especially is often challenged by severe fruit drop during the growth period, resulting in unstable yields [2,3]. Previous studies have shown that zinc, as an essential trace element for plants, participates in various enzymatic systems and auxin synthesis, playing a crucial role in enhancing fruit setting rate and improving fruit quality [4,5]. Therefore, exploring whether different types of zinc fertilizers can alleviate physiological fruit drop during blue honeysuckle growth and enhance yield stability holds significant practical importance for addressing current cultivation challenges.

In the growth and development of berry fruits, zinc plays a crucial role. It influences the efficiency of zinc utilization in plants by regulating and transporting zinc transporter proteins, thereby modulating plant growth and development [6]. Additionally, zinc is involved in the synthesis of plant hormones and the maintenance of ion transport, affecting plant growth and development by regulating growth hormone metabolism [4,5]. Research on strawberries has shown that zinc fertilizer application can significantly promote fruit yield, increasing it by 48.3% compared to the control (CK) [7]. Zinc treatments can also notably improve the external quality of strawberries, such as fruit firmness and fruit weight [8]. Studies on tomatoes have found that foliar application of zinc can rapidly supplement the nutrients required for tomato growth, thereby enhancing yield and improving fruit quality [9]. Zinc fertilizer can directly or indirectly affect the activity of carbohydrate-related enzymes at different developmental stages, regulating carbohydrate synthesis and consequently modulating sugar and acid content in apples [10,11]. Research indicates that zinc fertilizer treatment can increase the content of nutrients such as soluble solids, acidity, and ascorbic acid in citrus fruits [12]. Studies on grapes have found that foliar application of zinc fertilizer can significantly increase the soluble sugar content in the fruits [13], while zinc foliar spraying on pomegranates can significantly enhance the content of ascorbic acid and anthocyanins in the fruits [14]. This demonstrates that zinc fertilizer has a positive impact on fruit quality. For blue honeysuckle, changes in indicators such as soluble solids, ascorbic acid, and anthocyanin content directly affect its taste and nutritional value. Therefore, understanding how zinc fertilization influences these indicators is particularly important.

Currently, ZnSO_4_ and ZnCl_2_ are the most widely used zinc fertilizers in berry crops. However, excessive application of ZnSO_4_ can cause leaf burn and lead to nutrient imbalance. Research on citrus found that leaf necrosis spots can form when the foliar spray concentration exceeds 200 mg/L for ZnSO_4_ or 100 mg/L for ZnCl_2_ [15,16]. In contrast, ZnO nanoparticles (NPs), as a novel fertilizer produced via green synthesis, can significantly enhance zinc availability and utilization efficiency even at low concentrations [17]. Studies indicate that at equivalent concentrations, ZnO NPs exert a greater effect on fruit quality and yield compared to traditional zinc fertilizers. For example, strawberry yield and quality were significantly improved with ZnO NPs application relative to ZnSO_4_ [18]. Similarly, foliar application of 50 mg/L ZnO NPs on tomato seedlings significantly increased single fruit weight and total yield [19]. This is attributed to the high bioactivity of ZnO NPs. Their small size enables more effective penetration through biological barriers on plant surfaces, such as the leaf cuticle and stomata, thereby improving fertilizer absorption and utilization efficiency [20]. Furthermore, ZnO NPs offer other notable advantages, such as improving plant physiological and metabolic processes and enhancing tolerance to abiotic stress. For instance, foliar application of ZnO NPs on tomatoes can alleviate salt stress [21]. They can also reduce disease incidence by up to 50%, for example, by inhibiting the growth of the Botrytis cinerea pathogen in tomatoes [22].

While zinc fertilizers have been reported to enhance fruit quality and yield in other fruit crops, it is still unclear whether ZnO NPs can serve as an effective regulatory strategy to reduce blue honeysuckle fruit drop and stabilize quality under changing climatic conditions. Therefore, this study aims to investigate: (1) whether ZnO NPs offer superior advantages over traditional ionic zinc fertilizers in enhancing blue honeysuckle yield and fruit quality, and (2) how ZnO NPs of different particle sizes affect blue honeysuckle yield and fruit quality. Addressing these questions will not only provide an effective fertilization strategy to mitigate fruit drop and stabilize quality in blue honeysuckle cultivation under climate stress but also offer a theoretical foundation and practical guidance for the application of nano-fertilizers in specialty berry crops.

## 2. Results

### 2.1. Yield Analysis

**Figure 1:**
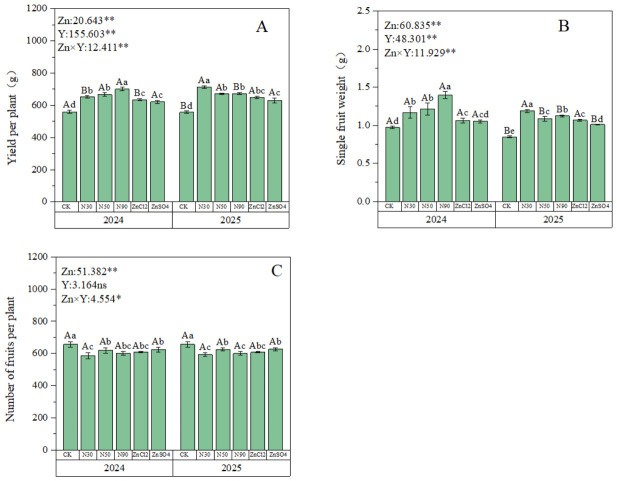
Effects of zinc fertilizer application on yield and quality of blue honeysuckle. (**A**) Impact of zinc fertilizer application on yield per plant. (**B**) Impact of zinc fertilizer application on fruit weight per plant. (**C**) Impact of zinc fertilizer application on fruit number per plant. Zn denotes different zinc fertilizer treatments, Y denotes interannual effects; numerical values represent F-values, where * indicates *p* < 0.05, ** and “ns” respectively indicate *p* < 0.01 significant difference and non-significant difference. Different lowercase letters indicate significant differences between Zn treatments (*p* < 0.05), while different uppercase letters represent significant differences between two-year treatments of the same Zn (*p* < 0.05).

Foliar zinc application significantly affected yield per plant and single fruit weight, but did not affect fruit quantity. These parameters were significantly influenced by year, and a significant interaction was observed between year and fertilizer treatment (Figure 1). The ZnO NPs outperformed zinc ions in yield per plant improvement, though the most effective treatment varied between years (Figure 1A). In 2024, 90 ZnO NPs showed the highest yield increase (25.4% above CK), while in 2025, 30 ZnO NPs achieved the best results (28.2% above CK) (Figure 1A). Regarding single fruit weight, the application of ZnO NPs in 2024 demonstrated significantly superior effects compared to zinc ions, with 90 ZnO NPs showing the best efficacy at 43.3% higher than CK (Figure 1B). In 2025, among all treatment groups, 30 ZnO NPs and 90 ZnO NPs exhibited significantly better results than other treatments, with 30 ZnO NPs achieving the highest efficacy at 40% higher than CK (Figure 1B). Zinc had no promoting effect on the fruit number of blue honeysuckles (Figure 1C). The results indicated that all treatments in both 2024 and 2025 were significantly lower than the control group, with minimal differences observed among treatments. Among these, 50 ZnO NPs and ZnSO_4_ showed the least inhibitory effect on fruit number.

### 2.2. External Fruit Quality Analysis

**Figure 2:**
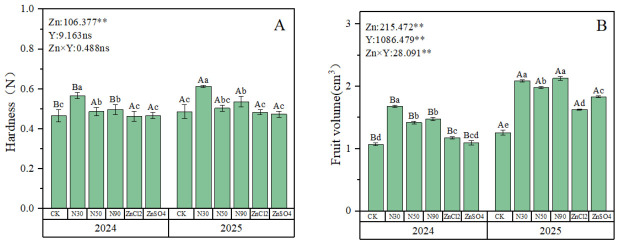
Effects of different zinc fertilizers on the external quality of blue honeysuckle. (**A**) Effects of different zinc fertilizers on fruit hardness. (**B**) Effects of different zinc fertilizers on fruit volume. Zn represents different zinc fertilizer treatments, Y represents interannual effects; numbers are F-values, where ** and “ns” respectively indicate significant differences (*p* < 0.01) and no significant difference. Different lowercase letters indicate significant differences between different Zn treatments (*p* < 0.05), while different uppercase letters represent significant differences between two-year treatments of the same Zn (*p* < 0.05).

Two-year data demonstrated that foliar zinc application significantly affected both fruit firmness and volume in blue honeysuckles. While fruit volume showed significant interannual variations, firmness exhibited no significant interannual differences. There was a significant interaction between year and fertilizer treatment on these indicators (Figure 2). Firmness results revealed that ZnO NPs treatments performed significantly better than zinc ions in both 2024 and 2025, with 90 ZnO NPs showing the most effective results in both years, achieving 21.5% and 26% higher firmness than the CK, respectively (Figure 2A). Regarding fruit volume, the application of ZnO NPs in both 2024 and 2025 demonstrated superior efficacy compared to ion zinc. In 2024, the optimal treatment for fruit volume was 30 ZnO NPs, showing a 56.8% increase over the CK. In 2025, the most effective treatments were 30 ZnO NPs and 90 ZnO NPs, with improvements of 65.8% and 69% respectively over the CK (Figure 2B).

### 2.3. Internal Fruit Quality Analysis

**Figure 3:**
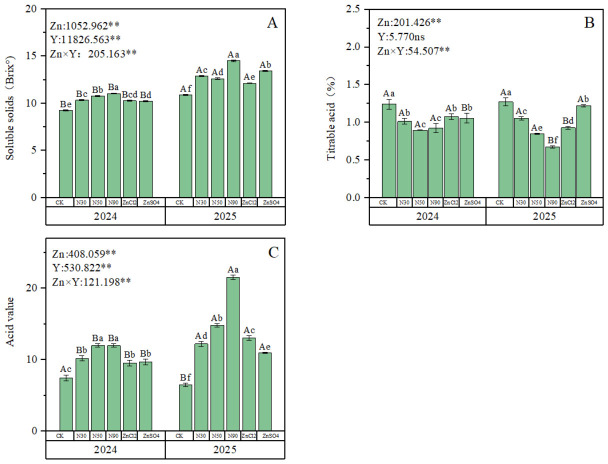
Effects of different zinc fertilizers on the internal quality of blue honeysuckle. (**A**) Effects of different zinc fertilizers on soluble solids in fruit shape. (**B**) Effects of different zinc fertilizers on titratable acids in fruits. (**C**) Effects of different zinc fertilizers on acid-to-solid ratio in fruits. Zn: represents different zinc fertilizer treatments, Y represents interannual effects; numbers are F-values, where ** and “ns” respectively indicate significant differences (*p* < 0.01) and no significant difference. Different lowercase letters indicate significant differences between Zn treatments (*p* < 0.05), while different uppercase letters represent significant differences between two-year treatments of the same Zn (*p* < 0.05).

Data from two years demonstrated that foliar zinc fertilizer application significantly affected soluble solids, titratable acid, and solid-to-acid ratio in blue honeysuckles, with significant interannual variations. There was a significant interaction between year and fertilizer treatment on these indicators (Figure 3). The soluble solids results showed that in 2024, treatments with 50 ZnO NPs and 90 ZnO NPs exhibited significantly better effects than other treatments, with 90 ZnO NPs showing the highest efficacy (19.1% higher than the CK, Figure 3A). In 2025, only the 90 ZnO NPs treatment demonstrated significantly superior effects compared to other treatments, exceeding the CK by 33.4% (Figure 3A). Lower titratable acid levels indicate reduced fruit acidity. The titratable acid results revealed that in both years, treatments with 50 ZnO NPs and 90 ZnO NPs significantly outperformed other treatments (Figure 3B). In 2024, the most effective treatments for titratable acid in fruits were 50 ZnO NPs and 90 ZnO NPs, with reduction rates of 27.9% and 25.7% below the CK, respectively. In 2025, the optimal treatment was 90 ZnO NPs, showing a reduction rate of 49.2% below the CK (Figure 3B). The solid-to-acid ratio treatment results indicated that in 2024, 50 ZnO NPs and 90 ZnO NPs significantly outperformed other treatment groups, with reduction rates of 60.8% and 60.4% above the CK, respectively. In 2025, 90 ZnO NPs demonstrated the most significant improvement, with a reduction rate of 231.5% above the CK (Figure 3C).

### 2.4. Nutrient Analysis

**Figure 4:**
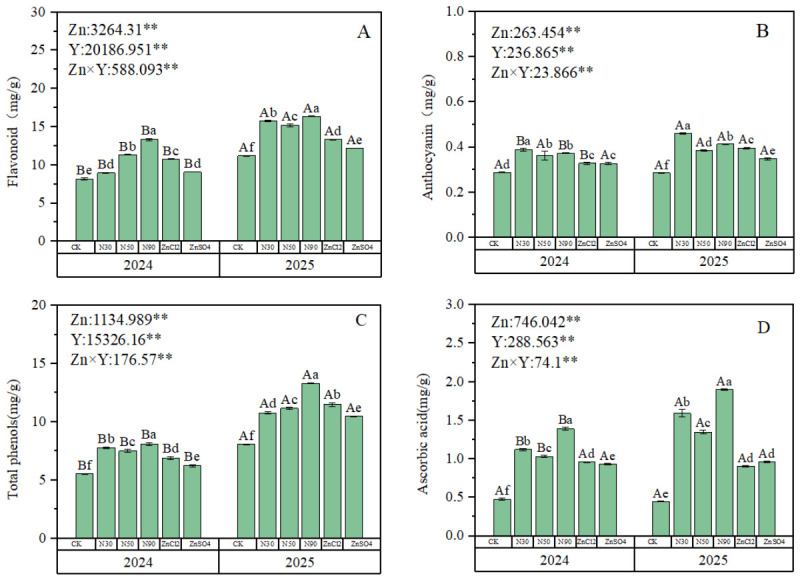
Effects of different zinc fertilizers on the internal quality of blue honeysuckle. (**A**) Effects of different zinc fertilizers on total flavonoid content in fruit shape. (**B**) Effects of different zinc fertilizers on anthocyanin content in fruits. (**C**) Effects of different zinc fertilizers on total phenolic content in fruits. (**D**) Effects of different zinc fertilizers on ascorbic acid content in fruits. Zn represents different zinc fertilizer treatments, Y represents interannual effects; numbers are F-values, where ** respectively indicate significant differences (*p* < 0.01). Different lowercase letters indicate significant differences between different Zn treatments (*p* < 0.05), while different uppercase letters represent significant differences between two-year treatments of the same Zn (*p* < 0.05).

Two-year data demonstrated that foliar zinc application significantly influenced total flavonoids, anthocyanins, total phenols, and ascorbic acid content in blue honeysuckle, with significant interannual variations. There was a significant interaction between year and fertilizer treatment on these indicators (Figure 4). For total flavonoids, 2024 treatment results showed that 50 ZnO NPs and 90 ZnO NPs treatments exhibited significantly better efficacy than other groups, with 90 ZnO NPs demonstrating the highest efficacy (62.7% higher than CK) (Figure 4A). In 2025, ZnO NPs treatment outperformed zinc ions, with 90 ZnO NPs showing the most significant improvement (46.2% higher than CK) (Figure 4A). The anthocyanin results demonstrated that in 2024, ZnO NPs treatment showed significantly superior efficacy compared to ion zinc, with 30 ZnO NPs exhibiting the best treatment effect, exceeding the CK by 34.8%. In 2025, both 30 ZnO NPs and 90 ZnO NPs treatment groups exhibited significantly better efficacy than other treatment groups, with 30 ZnO NPs again demonstrating the optimal treatment effect, surpassing the CK by 61% (Figure 4B). The total phenolic compounds treatment results demonstrated that in 2024, ZnO NPs exhibited significantly superior efficacy compared to zinc ions, with 90 ZnO NPs showing the best treatment effect, exceeding the CK by 45.7%. In 2025, the 90 ZnO NPs treatment group outperformed other treatment groups, surpassing the CK by 65.3% (Figure 4C). Ascorbic acid results indicated that in both 2024 and 2025, ZnO NPs treatment efficacy was significantly superior to zinc ions (Figure 4D), with 90 ZnO NPs again demonstrating the best treatment effect in both years, exceeding the CK by 191.7% and 323.8%, respectively (Figure 4D).

### 2.5. Comprehensive Evaluation Analysis of Radar Chart

**Figure 5:**
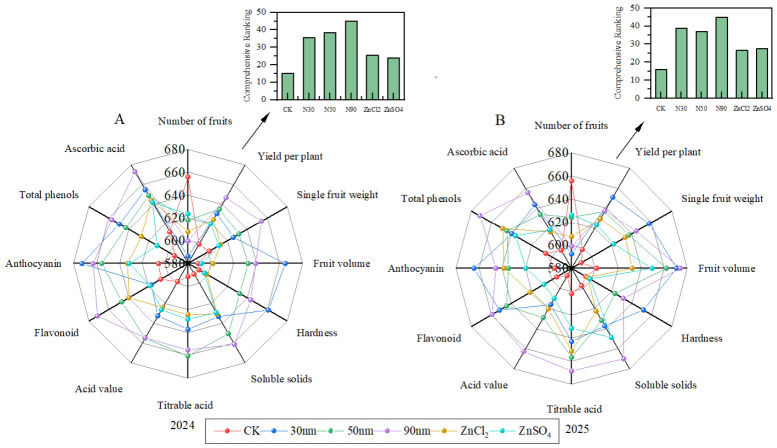
Radar chart of comprehensive evaluation analysis on foliar zinc fertilizer application for blue honeysuckle. (**A**) Radar analysis chart of various indicators under zinc fertilizer treatment in 2024. (**B**) Radar analysis chart of various indicators under zinc fertilizer treatment in 2025. The radar chart structure is arranged in sequence (clockwise) as follows: number of fruits per plant, yield per plant, fruit weight, fruit volume, hardness, soluble solids, titratable acid, solid-to-acid ratio, total flavonoids, anthocyanins, total phenols, and ascorbic acid. The bar chart is the comprehensive evaluation score of the radar chart.

The comprehensive evaluation analysis of zinc fertilizer foliar spraying on blue honeysuckles is shown in Figure 5. Analysis of all 12 evaluation subjects revealed significant differences in polygonal characteristics among treatment groups. As illustrated, the radar chart area of zinc-treated samples was larger than CK, indicating superior comprehensive indicators of blue honeysuckles under zinc treatment compared to the CK, and demonstrating a positive regulatory effect on blue honeysuckles. The radar analysis of the 2024 and 2025 growing years revealed similar radar chart profiles, indicating that zinc fertilizer had a stable effect on various indicators of blue honeysuckle, though differences persisted in some metrics. As shown in Figure 5A, 30 ZnO NPs demonstrated optimal effects on yield, single fruit weight, hardness, and anthocyanin content, whereas 90 ZnO NPs showed the most significant improvements in fruit volume, soluble solids, titratable acid, solid-to-acid ratio, flavonoids, total phenols, and ascorbic acid. Figure 5B further illustrates that 30 ZnO NPs excelled in fruit volume, hardness, and anthocyanin content, while 90 ZnO NPs outperformed in yield, single fruit weight, soluble solids, titratable acid, solid-to-acid ratio, flavonoids, total phenols, and ascorbic acid.

To better analyze the treatment effects of different zinc fertilizers, a comprehensive ranking of the five zinc fertilizers was conducted based on radar charts and plotted as bar graphs. As shown in the figure, the treatment effects of zinc fertilizers in 2024, from highest to lowest, were 90 ZnO NPs > 50 ZnO NPs > 30 ZnO NPs > ZnCl_2_ > ZnSO_4_ > CK; in 2025, the treatment effects of zinc fertilizers, from highest to lowest, were 90 ZnO NPs > 30 ZnO NPs > 50 ZnO NPs > ZnSO_4_ > ZnCl_2_ > CK.

## 3. Discussion

### 3.1. Nano-Zinc Fertilizer Enhances Yield and Quality in Blue Honeysuckle

Yield is one of the critical criteria for income generation and a key measure of a product’s economic value. Multiple studies have confirmed a positive correlation between berry plant yield and zinc fertilizer application [23,24]. This study demonstrated that two consecutive years of field trials on blue honeysuckle proved that spraying any form of zinc fertilizer significantly increased its yield. However, compared to the control (CK), the number of fruits decreased after zinc application. This suggests that the increase in fruit yield might be achieved by sacrificing fruit number in favor of promoting individual fruit weight (Figure 1). Research on tomatoes also indicates that zinc fertilizer primarily enhances total plant yield by increasing individual fruit weight [25], which aligns with the findings of this study (Figure 1). This because zinc fertilizer, as a key cofactor for various enzymes and the synthesis of auxin (IAA), promotes the growth of vegetative organs, which in turn leads to a decrease in fruit set, while the reduction in fruit number then promotes an increase in individual fruit weight [26]. Studies have found that zinc fertilizer significantly promotes cell division and expansion, thereby increasing fruit weight and improving fruit quality and total yield [27]. The more pronounced yield enhancement from ZnO NPs compared to ionic zinc is likely due to their stronger penetration ability. ZnO NPs can be more effectively assimilated and absorbed by leaves, increasing leaf zinc content, which subsequently exerts a positive influence on plant growth and yield increase [28,29]. This superior performance is mainly attributed to the fact that foliar-applied ZnO NPs promote the formation of photosynthetic pigments more effectively than ionic zinc (Figure S11), which helps increase total carbohydrate content, thereby promoting fruit growth and yield improvement [30,31,32].

In the present research, among all treatment groups, ZnO NPs exhibited the least inhibitory effect on fruit number (Figure 1C). Furthermore, foliar application of ZnO NPs outperformed ionic zinc treatments in terms of influencing yield-related parameters (yield, single fruit weight) in blue honeysuckle (Figure 1A). Similar results were reported by Monika et al., who found that ZnO NPs application provided the best improvement in fruit yield, fruit number, and single fruit weight compared to traditional ZnSO_4_ [18]. This is because the ultra-small size of zinc oxide nanoparticles allows them to be rapidly absorbed by plant cells, whereas ionic zinc often relies on aquaporins or ion transporters, resulting in a slower effect [33,34].

**Figure 6:**
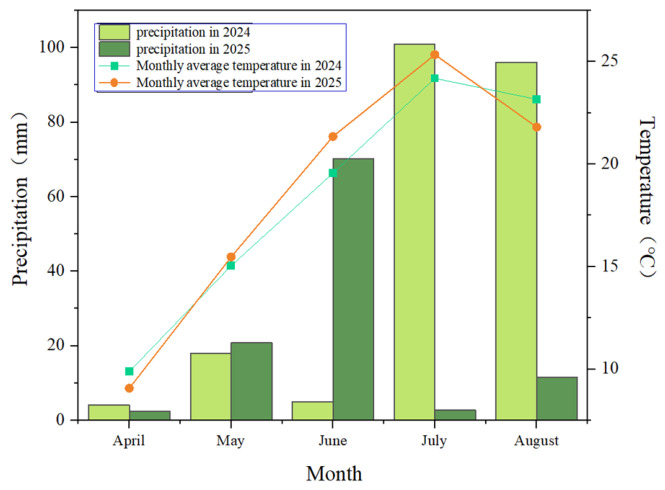
Temperature and precipitation from April to August in 2024 and 2025.

Furthermore, the effects of ZnO NPs with different particle sizes on yield-related parameters (yield per plant, single fruit weight, fruit number) varied. Based on integrated data from two-year field trials, regarding fruit number per plant, the 50 nm ZnO NPs treatment performed best in both years. For yield per plant and single fruit weight, the 90 nm ZnO NPs treatment showed the best results in 2024, while the 30 nm ZnO NPs treatment was most effective in 2025 (Figure 1A). The different promoting effects of nanoparticle size on yield over the two years were caused by variations in water availability across [35]. Analysis of temperature and precipitation data over two years revealed that the monthly average temperature during the fruiting period in 2024 was lower than in 2025. Additionally, precipitation in 2024 was predominantly concentrated in July and August, while rainfall during the primary fruiting period was relatively low. In contrast, precipitation in 2025 was mainly concentrated in June, which coincides with the critical growth stage of blue honeysuckle (Figure 6). Therefore, the 30 nm ZnO-NPs are small in size and can easily penetrate plant tissues through the stomata along with moisture, entering the interior of the plant [20,36].

External quality is a key visual factor influencing the sales and economic value of blue honeysuckle, primarily reflected by fruit size and firmness. Multiple studies have confirmed that zinc fertilizers have a positive effect on improving the external quality of berry fruits [8,37]. Zinc influences fruit firmness by participating in the metabolism of cell wall structural components, such as pectin and cellulose [38,39]. Furthermore, zinc can promote the synthesis of zinc finger proteins, including the zinc finger gene *SlPZF1*, which acts as a novel regulator of cell size during tomato fruit development. Its expression is crucial for fruit size control [40]. This study found that two-year field trials on blue honeysuckle demonstrated that spraying any form of zinc fertilizer significantly increased fruit size (Figure 2B and Figure S12). However, regarding fruit firmness, ZnO NPs showed a promoting effect, while ionic zinc fertilizers showed no significant difference compared to the control (CK) (Figure 2A). Previous research indicated that ionic zinc promotes fruit firmness at a concentration of 3000 mg/L [41]. In contrast, this study found that ZnO NPs at 80 mg/L enhanced fruit firmness, demonstrating their higher efficiency compared to ionic zinc. A similar conclusion was reached by Ahmed in a study on tomatoes, where foliar application of ZnO NPs at the same concentration outperformed ionic zinc fertilizers [27]. Additionally, the effects of different ZnO NP sizes on fruit size and firmness varied. Based on integrated data from two-year field trials, the 30 nm ZnO NPs treatment showed the best results for both fruit size and firmness.

### 3.2. Nano-Zinc Fertilizer Is More Effective in Flavor and Quality of Blue Honeysuckle

Soluble solids, titratable acidity, and the soluble solids-to-acidity ratio are important indicators affecting fruit taste [42]. Previous studies have found that zinc fertilizers can promote the improvement of fruit quality in berry plants [43,44]. This study showed that spraying zinc fertilizers significantly promoted the soluble solids content, titratable acidity, and soluble solids-to-acidity ratio of blue honeysuckle fruits, with the best treatment effect observed (Figure 3). This is because zinc can influence the activity of carbohydrate-related enzymes. Foliar application of zinc increases zinc content in the fruits, thereby enhancing carbohydrate metabolism and the activity of related enzymes, which in turn affects sugar accumulation in the fruits [45]. The effect of ZnO NPs spraying on the soluble solids-to-acidity ratio of blue honeysuckle was significantly better than that of ionic zinc treatments (Figure 3C). This finding is consistent with Monika’s study on strawberries, where foliar application of 200 mg/L ZnO NPs yielded the best results, significantly outperforming the 2000 mg/L ZnSO_4_ treatment group [18]. This is attributed to the ability of ZnO NPs to regulate plant photosynthesis, increase the translocation of photosynthetic products to the fruits, influence the balance between soluble solids and organic acids, and thereby improve the soluble solids-to-acidity ratio [46,47]. Furthermore, the effects of ZnO NPs with different particle sizes on soluble solids, titratable acidity, and the soluble solids-to-acidity ratio varied. Based on integrated data from two-year trials, for soluble solids content, the 90 nm ZnO NPs treatment yielded the best results in both years. Regarding titratable acidity, the 50 nm and 90 nm ZnO NPs treatments showed the best effects in 2024, while the 90 nm ZnO NPs treatment performed best in 2025. Overall, considering both years, the 90 nm ZnO NPs treatment demonstrated the best comprehensive effect.

Berry fruits are rich in various bioactive compounds, including anthocyanins, polyphenols, flavonoids, and ascorbic acid [48,49,50]. Numerous studies have found that zinc treatments significantly enhance the internal nutritional quality of fruits [51,52]. Consistent with previous research, this study observed that zinc fertilizer treatments significantly increased the content of nutrients (flavonoids, anthocyanins, total phenols, ascorbic acid) in the fruits (Figure 4). This is because zinc can activate antioxidant enzymes, thereby reducing the degradation of anthocyanins and flavonoids by reactive oxygen species [53,54]. Zinc fertilizer can also promote photosynthesis and berry development while influencing the expression of genes involved in the phenolic biosynthesis pathway, leading to an increase in phenolic compounds [44]. Furthermore, zinc enhances biofortification effects, improves the accumulation of mineral elements in plants, and consequently regulates fruit quality parameters such as ascorbic acid content [55].

Furthermore, based on flavonoid content from the two-year study, it was found that 90 ZnO NPs demonstrated optimal treatment efficacy, but there were significant differences in the effects of the 30 and 50 treatments on fruit quality between the two years. The 30 nm ZnO NPs treatment was less effective than ionic zinc in 2024, but in 2025, all ZnO NPs treatments were significantly more effective than ionic zinc. For anthocyanin content, all ZnO NPs significantly outperformed ionic zinc in 2024, but in 2025, the 50 nm ZnO NPs treatment was less effective than ionic zinc. Regarding total phenol content, all ZnO NPs were significantly superior to ionic zinc in 2024, whereas in 2025, only the 90 nm ZnO NPs treatment was significantly better than ionic zinc. This difference is primarily caused by environmental changes and cannot be fully mitigated by fertilization alone. Nevertheless, although blue honeysuckle fruit quality under identical zinc treatments differed significantly between the two years, the improvement in fruit quality following zinc treatment was markedly greater than that observed under the control and ionic zinc fertilizer treatments. This clearly demonstrates that nano-zinc fertilizer exhibits superior efficacy.

### 3.3. Comprehensive Effects Evaluation of Zinc Fertilizer Forms on Blue Honeysuckle

To more intuitively understand the comprehensive impact of different zinc fertilizer treatments on various indicators of blue honeysuckle, this study conducted an integrated analysis of 12 relevant indicators using a radar chart (Figure 5). The results showed that different zinc fertilizer treatments significantly promoted yield-related indicators (single fruit weight and yield per plant) of blue honeysuckle, but did not exhibit a clear promoting effect on fruit number. In terms of impact on yield indicators, ZnO NPs treatments outperformed ZnCl_2_ and ZnSO_4_. However, the effects of ZnO NPs with different particle sizes on yield indicators also varied. In 2024, 30 nm ZnO NPs showed the best effect on improving yield per plant and single fruit weight, while in 2025, 90 nm ZnO NPs performed best in enhancing single fruit weight and yield per plant. Integrating data from both years, we found that the impact of ZnO NP size on blue honeysuckle yield indicators was significantly influenced by interannual variations. This may be attributed to lower precipitation in May and June 2024 compared to 2025, which are critical periods for the growth and development of blue honeysuckle. Additionally, different ZnO NP sizes may interact with plant leaves through distinct mechanisms, potentially leading to significant variations in blue honeysuckle yield indicators.

An analysis of fruit quality indicators revealed that different zinc fertilizer treatments significantly promoted the fruit quality of blue honeysuckle (Figure 5). Furthermore, the effect of ZnO NPs was stronger than that of ZnCl_2_ and ZnSO_4_. It was also observed that the promoting effects of different ZnO NP sizes on quality indicators varied. This may be attributed to differences in the efficiency of action on leaves and the underlying mechanisms affecting fruit development among ZnO NPs of varying particle sizes, leading to distinct impacts on quality parameters. Regarding fruit firmness and anthocyanin content, the 30 nm ZnO NPs treatment yielded the best results in both years. For fruit size, the 30 nm ZnO NPs treatment was most effective in 2024, while both the 30 nm and 90 nm ZnO NPs treatments performed best in 2025. In contrast, for indicators including soluble solids, titratable acidity, soluble solids-to-acidity ratio, flavonoids, total phenols, and ascorbic acid, the 90 nm ZnO NPs treatment demonstrated the best results over the two-year period. These findings indicate that blue honeysuckle fruits treated with 30 nm ZnO NPs had firmer skin and larger size, but their overall quality was not optimal. In comparison, fruits treated with 90 nm ZnO NPs exhibited richer taste and higher nutritional value, albeit with softer skin.

To better analyze the effects of different zinc fertilizer treatments, a comprehensive ranking of the five zinc fertilizers (top three) was conducted based on the radar chart. In 2024, the treatment effectiveness from highest to lowest was 90 nm ZnO NPs > 50 nm ZnO NPs > 30 nm ZnO NPs. In 2025, the order was 90 nm ZnO NPs > 30 nm ZnO NPs > 50 nm ZnO NPs. Therefore, during the growth of blue honeysuckle, foliar application of 30 nm ZnO NPs is more effective in improving external quality and yield, making it more suitable for fresh-market sales. Application of 50 nm ZnO NPs shows intermediate effects across various indicators, providing a moderate promotion for all parameters. In contrast, foliar application of 90 nm ZnO NPs yields the best results in enhancing overall fruit quality, making it more suitable for processing and value-added product sales. Consequently, in blue honeysuckle cultivation, the appropriate zinc fertilizer can be selected based on specific production needs. Furthermore, for the large-scale application of ZnO-NPs in agricultural production, we evaluated their economic feasibility. The cost of ZnO-NPs was 0.41 yuan/mu, while that of ionic zinc was 1.02 yuan/mu (Table S1). The comparison shows that the cost of ZnO-NPs is lower than that of ionic zinc. Moreover, in actual production, the dosage of ZnO-NPs is significantly lower than that of ionic zinc, which can reduce environmental pollution while also delivering higher economic profits, demonstrating the feasibility of large-scale application.

## 4. Materials and Methods

### 4.1. Field Experiment

The experimental site was selected at the Xiangyang Base of Northeast Agricultural University, Harbin City, Heilongjiang Province (126°93′ E, 45°76′ N), which belongs to the temperate continental monsoon climate and is part of the first accumulated temperature zone in Heilongjiang Province. We conducted a two-year experiment at this site in 2024 and 2025. In 2024, rainfall was mainly concentrated in July and August, with low precipitation in June. The rainfall from April to August was 224.1 mm, and the annual average rainfall was 530 mm. The annual average temperature at the experimental site was 4.3 °C, with annual sunshine hours ranging from 2641 to 2732 h. The effective accumulated temperature (≥10 °C) was approximately 2300–2500 °C·d, and the frost-free period was 110–125 days. In 2025, rainfall was concentrated in June, with less precipitation in July and August. The rainfall from April to August was 107.5 mm (Figure 6), and the annual average rainfall was approximately 600 mm. The frost-free period was about 130–150 days, the annual average temperature was approximately 5.6 °C, and the annual sunshine hours ranged from 2460 to 2786 h. The effective accumulated temperature (≥10 °C) was approximately 2700–2900 °C·d. As a cold-climate crop, blue honeysuckle flowers in early spring and fruits in midsummer, with April to June being the primary growth period for blue honeysuckle development. The two-year temperature and precipitation data indicate that the monthly average temperature during the fruit growth period in 2024 was lower than that in 2025. In 2024, precipitation was mainly concentrated in July and August, with limited rainfall during the main fruit growth period. By contrast, precipitation in 2025 was primarily concentrated in June, a critical stage of fruit development.

Field management at the experimental site was as follows:

This study utilized 5-year-old blue honeysuckle plants, all propagated via green shoot cuttings, and planted on 5 June 2019. The standard spacing was 1 × 3.5 m. After autumn leaf fall, manual weeding and soil loosening were performed under the trees, with peat moss applied between rows to ensure root growth. Organic fertilizer was incorporated during autumn soil loosening, applied biennially. Apply urea (CO(NH_2_)_2_)(manufacturer: Henan Xinlianxin Chemical Industry Group Co., Ltd. (Henan China), (NH_4_)_2_HPO_4_ (manufacturer: Yunnan Yuntianhua Co.,Ltd. (Yunnan China), and microbial fertilizer in the ratio of 5:2:10 at the time of bud break (April 10) and fruit harvest (July 15) every year, with a single plant fertilization amount of 0.25 kg.

### 4.2. Experimental Design

This experiment employed a completely randomized block design with five treatments: Control (CK), 30 nm ZnO NPs (N30), 50 nm ZnO NPs (N50), 90 nm ZnO NPs (N90), ZnCl_2_, and ZnSO_4_. Treatments were administered during the young fruit, color-changing, and maturity stages, with a comparison between ZnO nanoparticles and ionic zinc sources. ZnO NPs (manufacturer: Shandong Keyuan Biochemical Co., Ltd. (Shangdong, China); purchaser: Shanghai Maclean Biochemical Technology Co., Ltd. (Shanghai, China); 30 nm particle size, catalog number: Z820774-100 g, batch number: C16250082; 50 nm particle size, catalog number: Z820772-100 g, batch number: C16412488; 90 nm particle size, catalog number: Z820773-100 g, batch number: C16507713). ZnCl_2_ from Tianjin Hengxing Chemical Reagent Manufacturing Co., Ltd. (Tianjin, China), and ZnSO_4_ from Tianjin Ruijinte Chemical Co., Ltd. (Tianjin, China). All reagents were at a concentration of 80 mg/L (through principal component analysis (PCA), 80 mg/L was identified as the optimal concentration for zinc fertilizer application, Figure S13). The concentrations were adjusted according to the optimal dosage of ZnO NPs, and all treatments were administered at the same concentration. Five plants with uniform growth conditions were selected for each treatment, and the experiment was repeated five times. The treatments were applied during the fruiting stage, fruit enlargement stage, and fruit coloring stage of the plants in 2024 and 2025, respectively. The treatments were sprayed onto the leaves using a pressure sprayer until the leaf surface was uniformly covered with the solution.

### 4.3. Sampling and Measurements

#### 4.3.1. Measurement of Yield per Plant and Fruit Number per Plant

In this study, fruits were harvested on 27 June 2024 and 18 June 2025. All fruits were collected in fruit collection boxes, with the number of fruits recorded and the yield measured using an electronic balance. After harvesting, 30 fruits were randomly selected from each plant for each parameter measurement, i.e., 150 fruits were harvested from each treatment and labeled. The fruits were stored in dry ice and transported back to the laboratory on the same day. Each treatment mixture was divided into two small samples, which were stored in 4 °C and −80 °C refrigerators, respectively.

#### 4.3.2. Determination Single-Fruit Weight and Fruit Volume

After the measurement, ten fruits were randomly selected, and the weight of each fruit was measured using an electronic balance, while the longitudinal and transverse diameters of the fruits were measured using a vernier caliper (model DL91150, Ningbo, China, accurate to 0.01 mm). The fruit volume was calculated according to Formula (1):

(1) $$Fruit\;volume=\frac{3.14}{6}\times transverse\;{diameter}^{2}\times vertical\;diameter$$

#### 4.3.3. Determination of Fruit Hardness

After the measurement, 10 fruits were randomly selected from each treatment and placed on the texture analyzer to measure the fruit hardness under different treatments.

#### 4.3.4. Determination of Soluble Solid Matter, Titratable Acid and Solid Acid Ratio in Fruit

After the measurement, the soluble solids (Brix) and titratable acid (%) content were determined using a handheld sugar-acid analyzer (Aito PAL–BXIACID-F5, Saitama, Japan). The solid-to-acid ratio was calculated according to Formula (2):

(2) $$Soild\;Acid\;Ratio=\frac{soluble\;solid}{titratable\;acid}$$

#### 4.3.5. Determination of Total Anthocyanin Content in Fruits

The total anthocyanin content was determined by the PH differential method [56] and calculated using Formulae (3) and (4):

(3) A=(A510−A700)PH1.0−(A510−A700)PH4.5

(4) $$Total\;anthocyanins\;content\left(mg/g\right)=\frac{A\times MW\times DF\times 1000}{\varepsilon \times m}$$

#### 4.3.6. Determination of the Total Phenolic Content in Fruits

The total phenol content was determined by the Folinol method [57], calculated according to Formulae (5) and (6):

(5) $$Standard\;curve:y=0.00496x-0.0027\left(R^{2}=0.9973\right)$$

(6) $$Total\;phenolic\;content\left(mg/g\right)=\frac{C\times V\times n}{m\times 1000}$$

#### 4.3.7. Determination of Total Flavonoid Content in Fruits

The total flavonoid content was determined by spectrophotometry [57] and calculated according to Formulae (7) and (8):

(7) $$Standard\;curve:y=5.2714x+0.0019\left(R^{2}=0.9993\right)$$

(8) $$Total\;flavonoids\;content\left(mg/g\right)=\frac{{m_{1}\times V}_{2}\times n}{m\times V_{1}}$$

#### 4.3.8. Determination of the Ascorbic Acid Content in Fruits

The ascorbic acid content was determined by the molybdenum blue colorimetric method [58], with the calculated values derived from Formulae (9) and (10):

(9) $$Standard\;curve:y=0.4085x+0.0404\left(R^{2}=0.9997\right)$$

(10) $$Ascorbic\;acid\;content\left(mg/g\right)=\frac{C\times \mathrm{Vt}\times n}{\mathrm{Vs}\times \mathrm{FW}}$$

### 4.4. Data Analysis

The normal distribution and homogeneity of variances for all the data were tested using the Waller–Duncan test with SPSS 22.0 software with Origin 2021 for data visualization. The effects of zinc fertilizer and interannual on yield indexes (yield per plant, fruit weight, fruit number, fruit hardness, fruit volume) and fruit quality (soluble solids, titratable acid, solid acid ratio, total flavonoids, anthocyanins, total phenols and ascorbic acid) of blue honeysuckle were evaluated by two-way ANOVA. When the interactions were significant, one-way ANOVA was used to evaluate the effects of a single factor by fixing the other factor, and significant differences in means were compared using Duncan’s multiple-comparison range test, with a significance level set at *p* < 0.05. The effects of zinc treatment on yield indexes and fruit quality and the effect of year on zinc treatment were analyzed by one factor analysis of variance. The correlation analysis further revealed the relationships between zinc treatment, interannual variations, and the yield and fruit quality of blue honeysuckle. Using Origin 2021 for radar chart analysis, a comprehensive evaluation was conducted on 12 indicators across two years of zinc treatment. The scoring rule was 1 point per grid, followed by ranking the cumulative scores of the 12 indicators to illustrate the effects of different zinc fertilizers on blue honeysuckle yield and fruit quality.

## 5. Conclusions

This study demonstrates that foliar zinc application enhances both yield and fruit quality in blue honeysuckle. At 80 mg/L concentrations, the 30 nm ZnO NPs treatment significantly increased fruit weight and per plant yield, while improving fruit hardness and anthocyanin content. Notably, the 90 nm ZnO NPs treatment showed superior internal quality parameters, including fruit volume, soluble solids, titratable acids, solid-to-acid ratio, total flavonoids, total phenols, and ascorbic acid levels, compared to other treatments. The results demonstrate that 30 nm ZnO NPs can stabilize and enhance production efficiency while improving the fruit firmness of blue honeysuckles, making them more suitable for storage and transportation. Meanwhile, 90 nm ZnO NPs significantly enhance the taste and nutritional quality of blue honeysuckles, making them more suitable for consumption and processing. These findings provide a novel approach to increasing blue honeysuckle yield and improving fruit quality, offering a theoretical basis for their practical application in production.

However, several limitations should be acknowledged. The conclusion that ZnO NPs are superior to ionic zinc fertilizers is based solely on comparisons at a single concentration (80 mg/L). The relative efficacy of these zinc forms may vary across different concentration ranges. Second, due to sample depletion after the two-year field experiment and biochemical analyses, we were unable to perform independent characterization of the ZnO NPs using TEM and DLS. While supplier-provided specifications and the literature on similar nanoparticles are referenced, the lack of in-house characterization constitutes a technical limitation. Future studies should include rigorous characterization of nanomaterials prior to application and conduct full concentration-response analyses.

## Data Availability

The raw data supporting the conclusions of this article will be made available by the authors on request.

## Supplementary Materials

The following supporting information can be downloaded at: https://www.mdpi.com/article/10.3390/plants15101520/s1. Table S1: Cost Comparison Table of Nano Zinc Oxide and Ionic Zinc; Figure S1: The effect of foliar application of different zinc fertilizers on plant height; Figure S2: Effect of foliar spraying with different concentrations of zinc fertilizer on leaf SPAD; Figure S3: Effects of Different Zinc Fertilizer on Zinc Content in Leaves of Blue Honeysuckle; Figure S4: Effects of Different Zinc Fertilizer on Zinc Content in Fruit of Blue Honeysuckle; Figure S5: Effects of Spraying Different Zinc Fertilizers on Soluble Sugar Content in Fruits; Figure S6: Effects of Different Zinc Fertilizers on SOD Content in Fruits by Spraying Application; Figure S7: Effects of Different Zinc Fertilizers on POD Content in Fruits by Spraying Application; Figure S8: Effects of Different Zinc Fertilizers on Fruit CAT Content by Spraying Application; Figure S9: Effects of Spraying Different Zinc Fertilizers on MDA Content in Fruits; Figure S10: Effects of Different Zinc Fertilizers on Fruit PPO Content by Spraying Application; Figure S11: Effects of Different Zinc Fertilizers on APX Content in Fruits by Spraying Application; Figure S12: Fruit morphology under different zinc fertilizer treatments; Figure S13: A: PCA of Zinc Fertilizer Foliar Application Treatment in 2024. B: PCA of Zinc Fertilizer Foliar Application Treatment in 2025.
