# Supplementary material for: Morphological Advantages of Nano-Zinc: Effects on Yield and Quality Improvement in Blue Honeysuckle
Source: Plants (Basel). 2026 May 15;15(10):1520. doi: 10.3390/plants15101520 (PMC13210828; doi:10.3390/plants15101520)
Supplement: Supplementary file 1 [file plants-15-01520-s001.zip › plants-4247490-supplementary.pdf]

Supplementary Data

Table S1. Cost Comparison Table of Nano Zinc Oxide and Ionic Zinc.

	ZnO NPs	Zinc ion
Vendor	Shaanxi Sino Academy Nano Materials Co.,Ltd.	Bohigh Zinc Product Co.,Ltd.
Unit price (yuan/ton)	23000	2300
Application rate (g/mu)	18	444
Cost (yuan/mu)	0.41	1.02

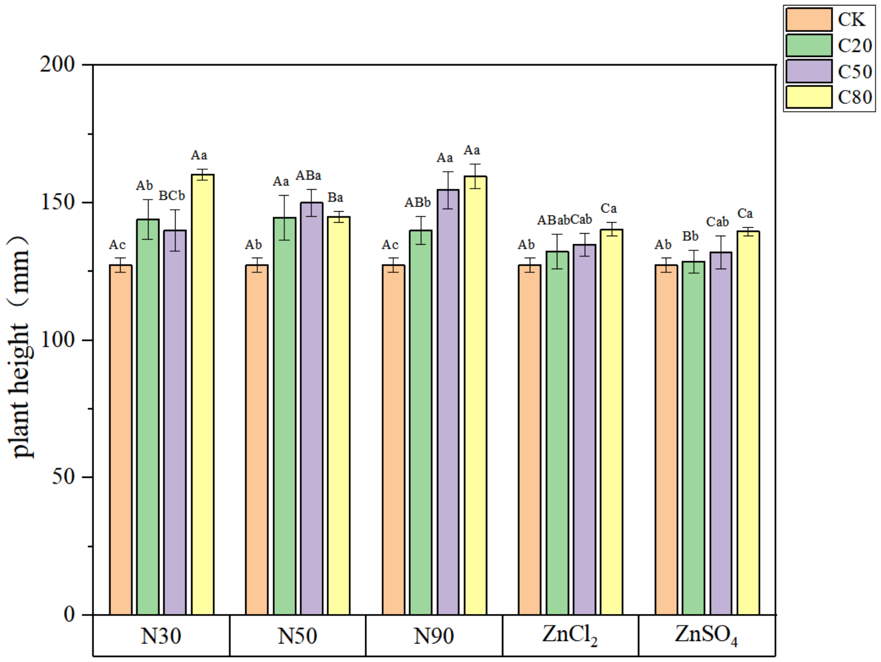

Figure S1. The effect of foliar application of different zinc fertilizers on plant height. N represents nanoparticle size; CK: 0 mg/L(treated with water), C20: 20 mg/L, C50: 50 mg/L, C80: 80 mg/L. Different lowercase letters indicate significant differences between treatments with different concentrations of the same zinc fertilizer ($P < 0.05$); different uppercase letters represent significant differences between treatments with the same concentration but different zinc fertilizers ($P < 0.05$).

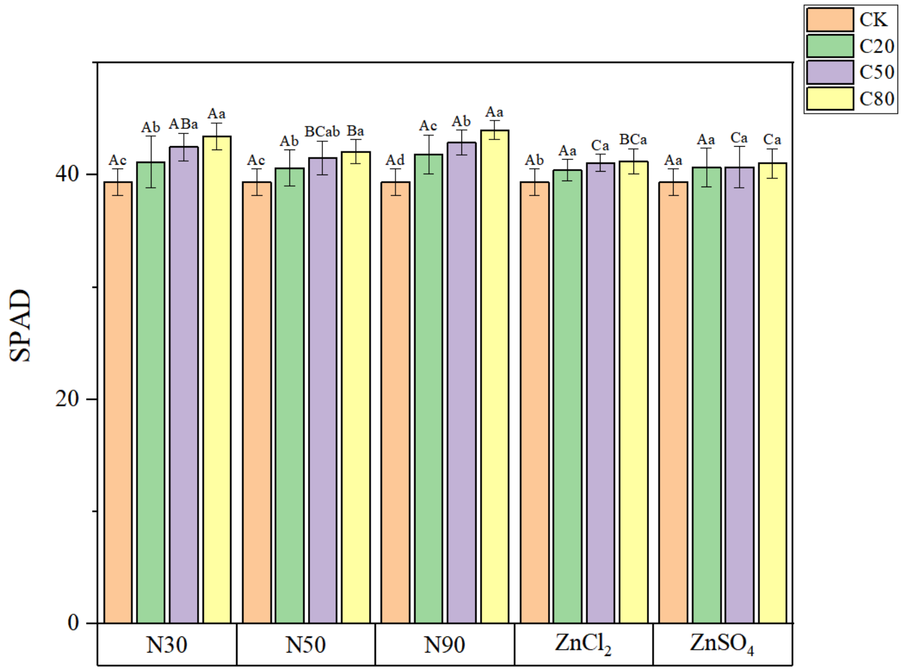

Figure S2. Effect of foliar spraying with different concentrations of zinc fertilizer on leaf SPAD. N represents nanoparticle size; CK: 0 mg/L(treated with water), C20: 20 mg/L, C50: 50 mg/L, C80: 80 mg/L. Different lowercase letters indicate significant differences between treatments with different concentrations of the same zinc fertilizer ($P < 0.05$); different uppercase letters represent significant differences between treatments with the same concentration but different zinc fertilizers ($P < 0.05$).

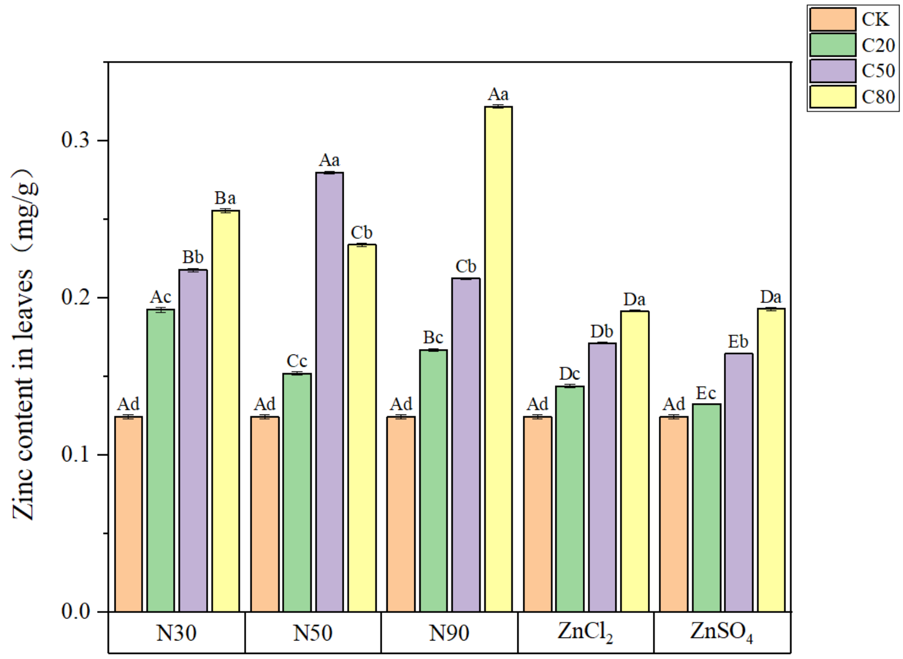

Figure S3. Effects of Different Zinc Fertilizer on Zinc Content in Leaves of Blue Honeysuckle. N represents nanoparticle size; CK: 0 mg/L(treated with water), C20: 20 mg/L, C50: 50 mg/L, C80: 80 mg/L. Different lowercase letters indicate significant differences between treatments with different concentrations of the same zinc fertilizer ($P < 0.05$); different uppercase letters represent significant differences between treatments with the same concentration but different zinc fertilizers ($P < 0.05$).

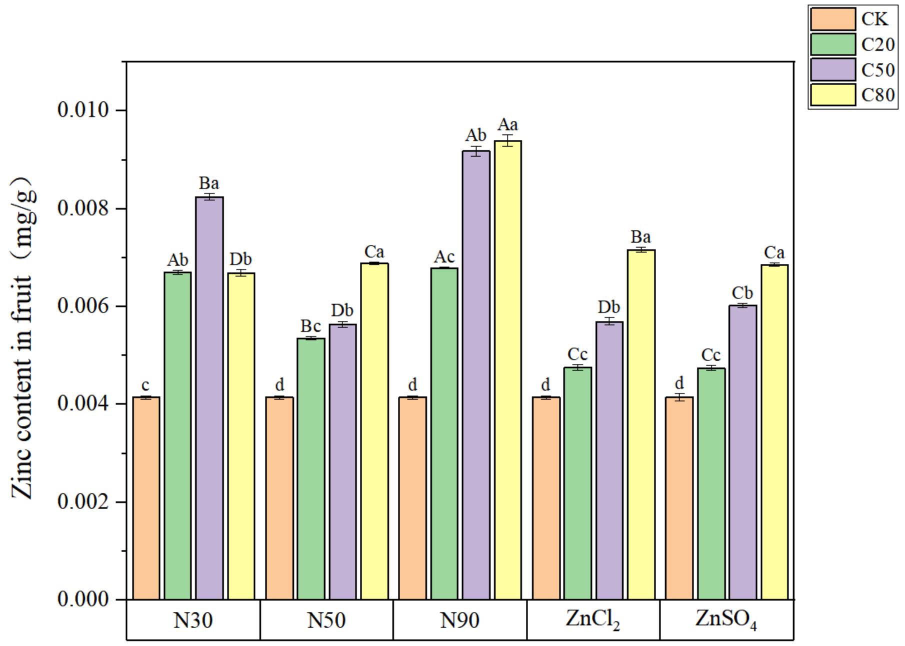

Figure S4. Effects of Different Zinc Fertilizer on Zinc Content in Fruit of Blue Honeysuckle. N represents nanoparticle size; CK: 0 mg/L(treated with water), C20: 20 mg/L, C50: 50 mg/L, C80: 80 mg/L. Different lowercase letters indicate significant differences between treatments with different concentrations of the same zinc fertilizer (P <0.05); different uppercase letters represent significant differences between treatments with the same concentration but different zinc fertilizers (P <0.05).

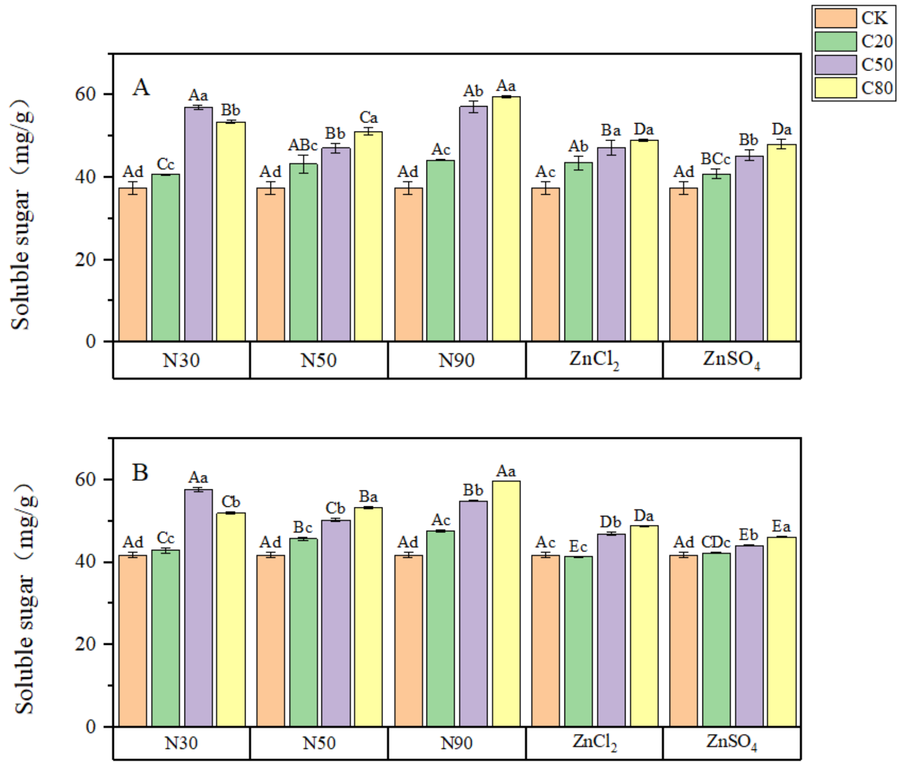

Figure S5. Effects of Spraying Different Zinc Fertilizers on Soluble Sugar Content in Fruits. N represents nanoparticle size; CK: 0 mg/L(treated with water), C20: 20 mg/L, C50: 50 mg/L, C80: 80 mg/L. Effect of different zinc fertilizers on soluble sugar content in Blue honeysuckle in 2024; B Effect of different zinc fertilizers on soluble sugar content in Blue honeysuckle in 2025. Different lowercase letters indicate significant differences between treatments with different concentrations of the same zinc fertilizer (P <0.05); different uppercase letters represent significant differences between treatments with the same concentration but different zinc fertilizers (P <0.05).

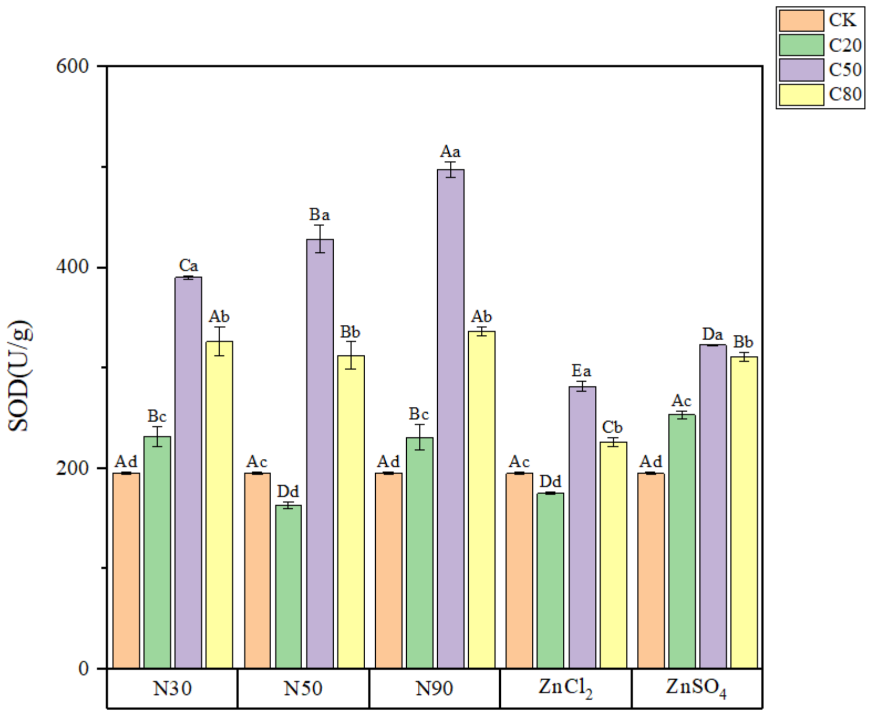

Figure S6. Effects of Different Zinc Fertilizers on SOD Content in Fruits by Spraying Application. N represents nanoparticle size; CK: 0 mg/L(treated with water), C20: 20 mg/L, C50: 50 mg/L, C80: 80 mg/L. Different lowercase letters indicate significant differences between treatments with different concentrations of the same zinc fertilizer ($P < 0.05$); different uppercase letters represent significant differences between treatments with the same concentration but different zinc fertilizers ($P < 0.05$).

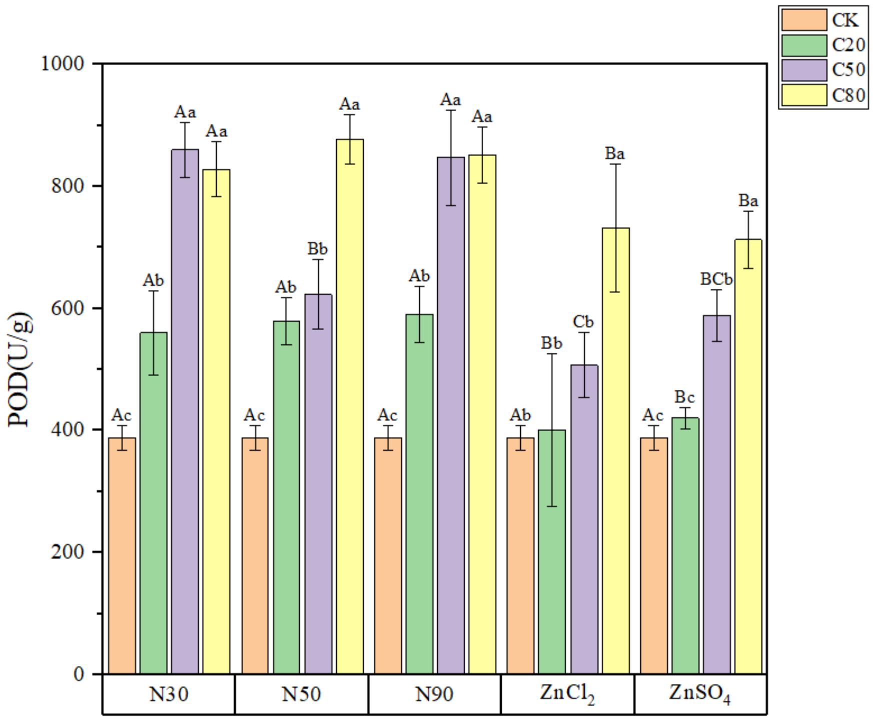

Figure S7. Effects of Different Zinc Fertilizers on POD Content in Fruits by Spraying Application. N represents nanoparticle size; CK: 0 mg/L(treated with water), C20: 20 mg/L, C50: 50 mg/L, C80: 80 mg/L. Different lowercase letters indicate significant differences between treatments with different concentrations of the same zinc fertilizer ($P < 0.05$); different uppercase letters represent significant differences between treatments with the same concentration but different zinc fertilizers ($P < 0.05$).

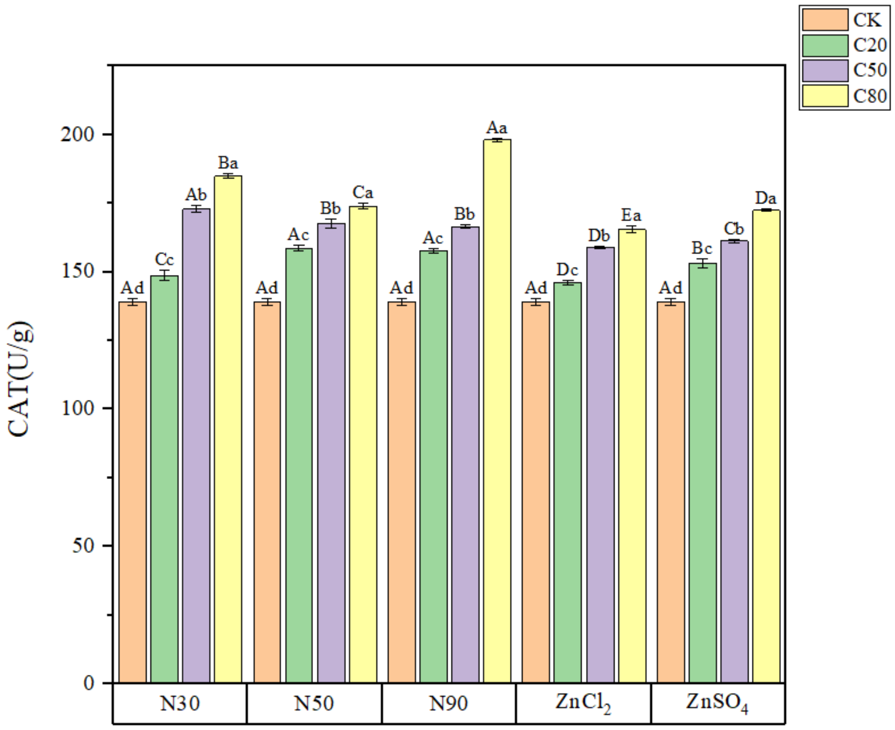

Figure S8. Effects of Different Zinc Fertilizers on Fruit CAT Content by Spraying Application. N represents nanoparticle size; CK: 0 mg/L(treated with water), C20: 20 mg/L, C50: 50 mg/L, C80: 80 mg/L. Different lowercase letters indicate significant differences between treatments with different concentrations of the same zinc fertilizer ($P < 0.05$); different uppercase letters represent significant differences between treatments with the same concentration but different zinc fertilizers ($P < 0.05$).

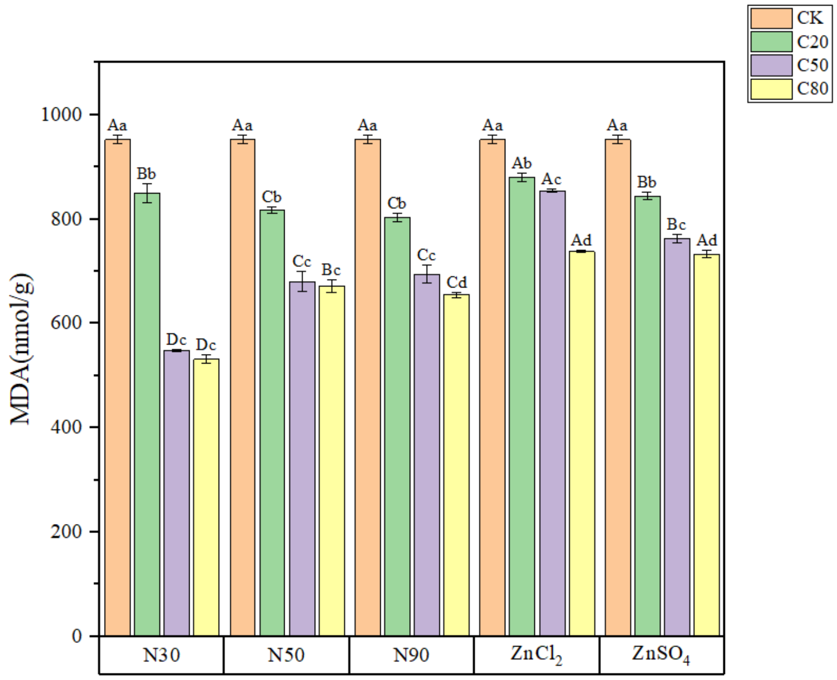

Figure S9. Effects of Spraying Different Zinc Fertilizers on MDA Content in Fruits. N represents nanoparticle size; CK: 0 mg/L(treated with water), C20: 20 mg/L, C50: 50 mg/L, C80: 80 mg/L. Different lowercase letters indicate significant differences between treatments with different concentrations of the same zinc fertilizer ($P < 0.05$); different uppercase letters represent significant differences between treatments with the same concentration but different zinc fertilizers ($P < 0.05$).

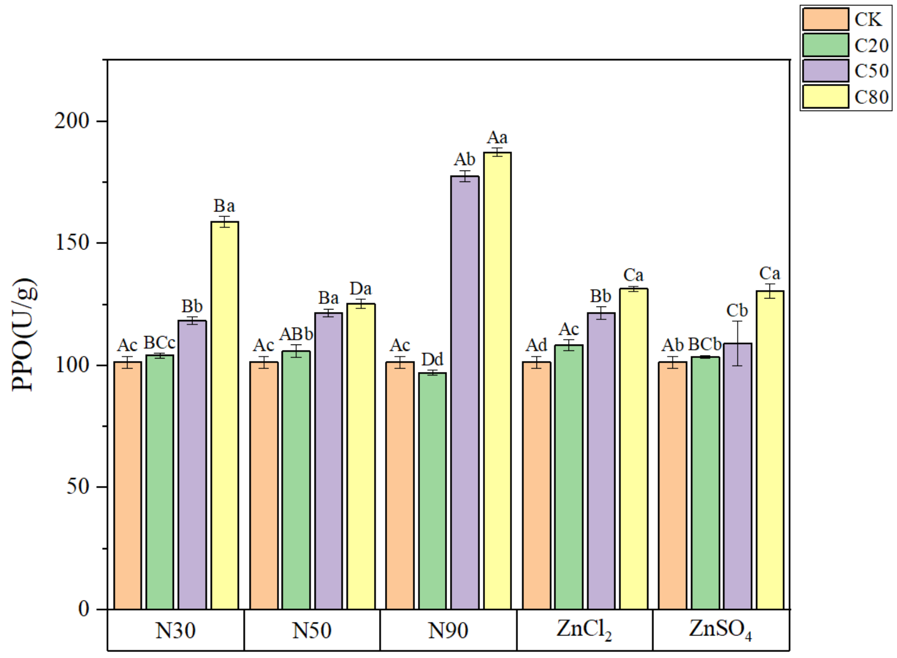

Figure S10. Effects of Different Zinc Fertilizers on Fruit PPO Content by Spraying Application. N represents nanoparticle size; CK: 0 mg/L(treated with water), C20: 20 mg/L, C50: 50 mg/L, C80: 80 mg/L. Different lowercase letters indicate significant differences between treatments with different concentrations of the same zinc fertilizer (P <0.05); different uppercase letters represent significant differences between treatments with the same concentration but different zinc fertilizers (P <0.05).

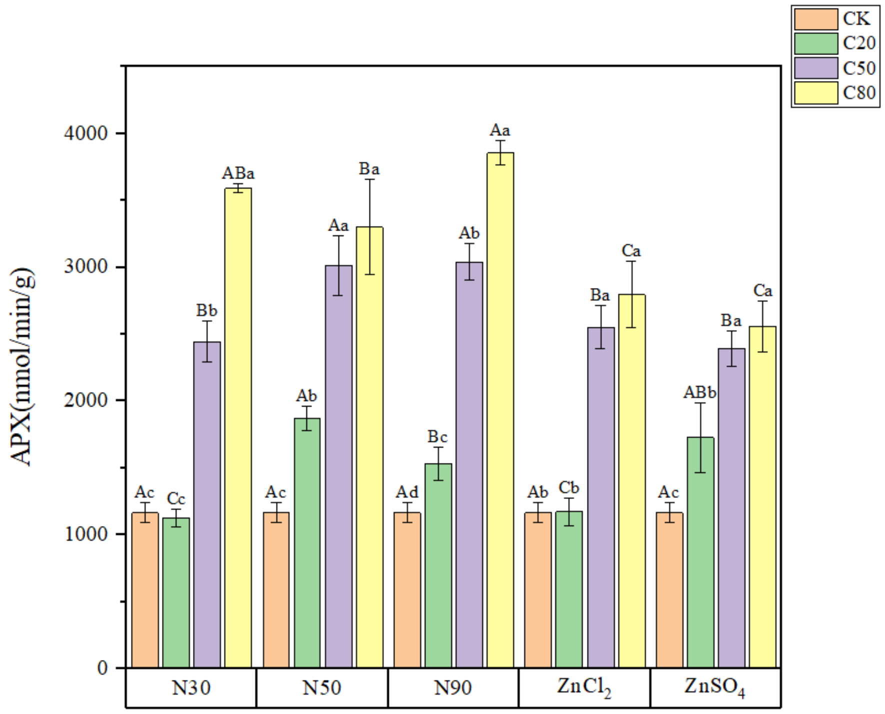

Figure S11. Effects of Different Zinc Fertilizers on APX Content in Fruits by Spraying Application. N represents nanoparticle size; CK: 0 mg/L(treated with water), C20: 20 mg/L, C50: 50 mg/L, C80: 80 mg/L. Different lowercase letters indicate significant differences between treatments with different concentrations of the same zinc fertilizer (P <0.05); different uppercase letters represent significant differences between treatments with the same concentration but different zinc fertilizers (P <0.05).

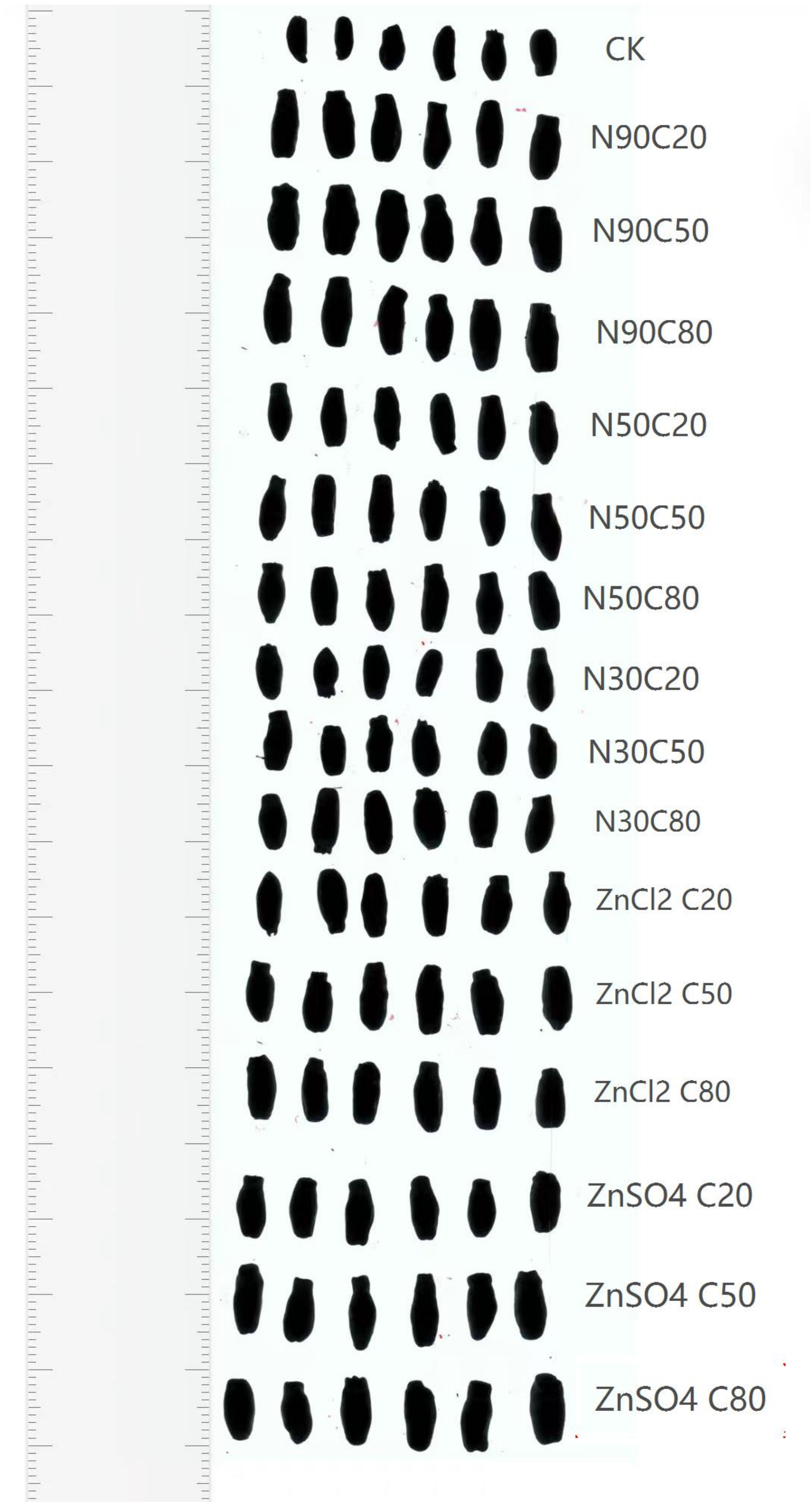

Figure S12. Fruit morphology under different zinc fertilizer treatments. N represents nanoparticle size; C represents concentration. CK: 0 mg/L(treated with water), C20: 20 mg/L, C50: 50 mg/L, C80: 80 mg/L.

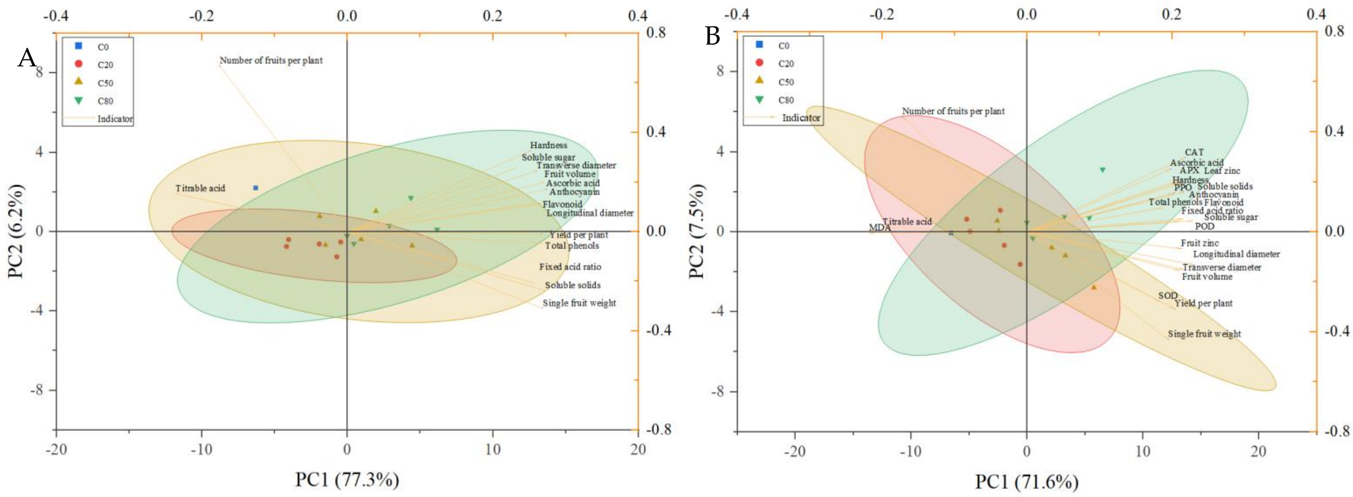

Figure S13. A: PCA of Zinc Fertilizer Foliar Application Treatment in 2024. B: PCA of Zinc Fertilizer Foliar Application Treatment in 2025. C0: 0 mg/L(treated with water), C20: 20 mg/L, C50: 50 mg/L, C80: 80 mg/L. The biplot revealed that samples from the control (C0) and C20 treatments were predominantly clustered in the negative region of PC1, while C50-treated samples exhibited a transitional distribution across the axis. In contrast, C80-treated samples shifted markedly toward the positive region of PC1, indicating a clear separation between the effects of high-concentration zinc treatment and those of the control and low-concentration treatments. Figure A: PC1 and PC2 accounted for 77.3% and 6.2% of the variance, respectively, with a cumulative contribution rate of 83.5%. Indicators such as fruit firmness, soluble solids, transverse diameter, longitudinal diameter, fruit volume, ascorbic acid, anthocyanins, and total phenolics were all associated with the C80 treatment and showed a positive correlation with PC1. Figure B: PC1 and PC2 explained 71.6% and 7.5% of the variance, respectively, with a cumulative contribution rate of 79.1%. Indicators including fruit firmness, soluble sugar, soluble solids, solid-acid ratio, ascorbic acid, anthocyanins, flavonoids, total phenolics, leaf zinc content, and the activities of CAT, APX, POD, and PPO were all aligned with the C80 treatment and exhibited a significant positive correlation with PC1. In summary, zinc treatment synergistically enhanced fruit yield and quality by improving fruit antioxidant enzyme activities and nutritional properties, with the C80 treatment demonstrating the most pronounced overall effect.
